# Comparison of average daily gain, apparent digestibility, rumen fermentation parameters and bacterial communities, and serum antioxidant indices in Leizhou goats fed with or without rumen-protected fat

**DOI:** 10.3389/fvets.2024.1518826

**Published:** 2024-12-23

**Authors:** Hu Liu, Hongxiang Mao, Wenji Wang, Weishi Peng, Kaiyu Mao, Wei Sun, Yuanting Yang, Qun Wu, Ke Wang, Meng Zeng, Jiancheng Han, Hanlin Zhou

**Affiliations:** ^1^Zhanjiang Experimental Station, Chinese Academy of Tropical Agricultural Sciences, Zhanjiang, China; ^2^Wilmar (Shanghai) Biotechnology Research & Development Center Co., Ltd., Tianjin, China; ^3^Department of Animal and Veterinary Sciences, AU Viborg-Research Center Foulum, Aarhus University, Tjele, Denmark; ^4^College of Animal Science and Technology, Guangxi University, Nanning, Guangxi, China; ^5^College of Animal Science and Technology, International Joint Research Laboratory in Universities of Jiangsu Province of China for Domestic Animal Germplasm Resources and Genetic Improvement, Yangzhou University, Yangzhou, China; ^6^Sanya Research Institute, Chinese Academy of Tropical Agricultural Sciences, Sanya, China

**Keywords:** rumen protected fat, goats, average daily gain, rumen bacteria communities, apparent total tract digestibilities

## Abstract

**Introduction:**

Rumen-protected fat (RPF) is a vital dietary energy source for dairy cows. However, the influences of RPF on rumen volatile fatty acid (VFA) content and bacterial communities in goats are poorly documented.

**Methods:**

In this study, 12 castrated male goats (body weight [BW]: 13.3 ± 0.02 kg; 6 months of age) were used as the experimental animal and then allocated into two groups (*n* = 6): a control group, fed a basal diet without RPF supplementation, and the RPF supplementation group, fed a basal diet with 2.4% RPF supplementation.

**Results:**

The final BW and ADG were higher (*p* < 0.05) and the ratio of dry matter (DM) intake to ADG was lower (*p* < 0.05) in the 2.4% RPF supplementation group compared with those in the control group. The 2.4% RPF supplementation group showed a higher total tract digestibility of DM, CP, OM, neutral and acid detergent fiber compared with that of the control group (*p* < 0.05). The proportion of acetate was higher (*p* < 0.05) and that of propionate was lower (*p* < 0.05) in the 2.4% RPF supplementation group compared with those in the control group. The relative abundances of *Christensenellaceae_R-7_group*, *unclassified_f__Selenomonadaceae*, *norank_f__Selenomonadaceae*, *Quinella*, *norank_f__Bacteroidales_RF16_group*, and *unclassified_o__Bacteroidales* were higher (*p* < 0.05) and those of *Lachnospiraceae_NK3A20_group*, *norank_f__F082*, *Olsenella*, *Erysipelatoclostridiaceae_UCG-004*, and *Syntrophococcus* were lower (*p* < 0.05) in the 2.4% RPF supplementation group compared with those in the control group.

**Discussion:**

In conclusion, 2.4% RPF supplementation can improve the ADG and antioxidant capacity by regulating the rumen bacterial communities and enhancing the apparent total tract digestibility in growing Leizhou goats.

## 1 Introduction

Ensuring food security is a critical global objective. Ruminants, mainly goats, sheep, dairy cows, and cattle, are essential for ensuring food security because they provide meat and milk from non-edible fibrous biomass. In China, there are numerous types of non-edible fibrous biomass that could be used as forage by ruminants. However, the efficiency of ruminant production in China is very slow, and the proportion of meat from ruminants consumed only accounts for up to 13.5% of the total meat consumption (1, 2). Hence, currently, there is an urgent need to improve ruminant average daily gain.

In China, there are 79 goat breeds with a total population of approximately 130 million (3). The indigenous Leizhou goats (*Capra hircus L*), also known as Hainan Black goats, are a local breed renowned for their flavorful meat Currently, they are mainly raised on the Leizhou Peninsula and Hainan Island in the south of China, with tropical climate and the precipitation was 1,640 mm, and are grazed throughout the year (3). However, the productive efficiency of goats is low, and their meat yield cannot meet the requirements of human consumption.

The rumen is a vital digestive organ, which inhabited numerous microorganisms and provided energy and protein (4, 5). Rumen-protected fats (RPF) are regarded as a vital energy source for ruminants because they avoid fat degradation by rumen microbes, which allows them to be utilized by ruminants when they into the small intestine. The RPF can increase the average daily gain (ADG) in goats (6) and Korean Native steers (7). In addition, total tract digestibility is increased in sheep that consume a diet containing calcium soap and palm fatty acids (4). In addition, at phylum levels in the cerum bacterial communitie, the Firmicutes increased, whereas the Bacteroidota and Desulfobacterota decreased with supplementation of RPF; at the gens levels, RPF supplementation increased the abundances of *Christensenellaceae_R-7_group*, [*Eubacterium*]*_coprostanoligenes_group_unclassified*, *Lachnospiraceae_unclassified* and *Ruminococcaceae_unclassified* in the cerum sample of the Saanen goats (8). Also, the serum metabolisms indexes could change, for example, the leptin were decreased when the beef cattle consumed a dietary containing RPF (9). A previous study reported that RPF supplementation 30 g/d per head could enhance the growth performance in finishing goats (10). Moreover, supplementation with RPF can enhance energy efficiency because the medium- or long-chain fatty acid composition of fat can decrease methane production in ruminants (11, 12). Together, these effects can lead to improved growth performance in goats and cattle.

In our previous study, the RPF could enhance the growth performance of Leizhou goats by regulating their fecal bacteria communities (6). However, the apparent digestibility of nutrients and the rumen fermentation traits and bacterial communities is still unclearly. Therefore, we hypothesized that RPF supplementation can increase growth performance by improving nutrient digestion and altering the ruminal bacterial community in goats. To test our hypothesis, ADG, apparent total tract nutrient digestibility, rumen volatile fatty acids (VFAs), ruminal nitrogen metabolism, bacterial communities, and serum metabolite indices were measured in goats fed RPF.

## 2 Materials and methods

This study conducted from October to December 2023 at Zhanjiang Experimental Station (ZES), Chinese Academy of Tropical Agricultural Sciences (CATAS), Zhanjiang City, Guangdong Province, China (21°16′12″ N, 110°21′27″ E). All experiments procedures were performed following the guidelines of the Animal Ethics Committee of ZES, CATAS (protocol no. ZES 202306010).

### 2.1 Goats, diets, and experimental design

The management strategy for the experimental goats and the design of the experiment were based on those described in our previous study (6). Briefly, 12 castrated growing Leizhou goats (average body weight of 13.3 ± 0.02 kg) with an average age of 6 months were selected. These goats were assigned randomly into two treatment groups: a control diet (CON) without RPF and control diet containing 2.4% RPF groups. The RPF products consisted of individual fatty acids, mainly C16:0 (480 g/kg), C18:0 (50 g/kg), C18:1 (360 g/kg), C18:2 (90 g/kg), and C14:0 (20 g/kg). The RPF supplementation level followed the recommendations of the Yihai Kerry Arawana Holdings Co., Ltd. (Shanghai, China). The composition and nutritional levels of the control diet were the same as those described by Liu et al. and NY/T 816–2021 (Table 1) (6, 13). The RPF is additional addition in the dietary. All RPF was incorporated into the basal dietary in the morning feeding daily (0800 h) and every goat in the RPF groups was consumed. All goats had *ad libitum* access to feed and water.

**Table 1:** Ingredients and chemical composition of the feed provided to the Leizhou goats.

Items	Content
Ingredients, g/kg of feed	
Corn straw	500
Corn	150
Soybean meal	130
Barley grain	80.0
Wheat bran	50.0
DDGS	41.5
Sodium bicarbonate	10.0
Limestone	12.0
Calcium hydrogen phosphate	9.50
Sodium chloride	5.00
Urea	2.00
Premix^1^	10.0
Chemical composition, g/kg DM	
Dry matter	915
Crude protein^2^	135
Ether extract	50.0
Neutral detergent fiber	410
Acid detergent fiber	235
Calcium	7.6
Phosphorus	3.2
Metabolizable energy^3^, MJ/kg	8.00

DDGS, distillers dired grains with solubles.

1 The premix comprised the following per kg: vitamin D 2000 IU, vitamin A 12000 IU, vitamin E 30 IU, Fe 64 mg, Cu 12 mg, Zn 60 mg, Mn 56 mg, Se 0.35 mg, I 1.2 mg, Co 0.4 mg.

2 Calculated as concentration of nitrogen × 6.25.

3 Calculated values.

The goats were allowed a 14-day adaptation period to the experimental diets in individual cages, and then followed by a 42-day experimental period to collect growth performance data and feed, residual feed, fecal, rumen fluid, and serum samples. Feed was weighed daily and provided to the goats two times at 0800 h and 1700 h, respectively. Residual feed was collected and weighed daily before 0800 h, and DM intake was calculated as the feed provided daily minus the residual feed, at 0800 h, throughout the experimental data and sample collection period. Goats were weighed at first day and the end of the 42-day experimental period before 0800 h, and the ADG (g/d) was calculated by dividing the BW gain (Final BW–initial BW) over the 42 days. Feed conversion efficiency was obtained by dividing the ADG (g) by the average daily DM intake (g).

### 2.2 Experimental procedures and samples collection

The digestibility experiment was carried out for a four consecutive days from days 39 to 42. Approximately 80 g/d of feed and residual feed were collected on day 39 to 42. Fecal samples (>20 g per sampling) were collected at 0000, 0600, 1,200, and 1800 h daily, following the methods described by Lourenco et al. (14). Feed, residual feed, and fecal samples were kept in separate Ziplock bags (160 mm × 280 mm; product number: 19250; Deli Company, Ningbo City, China). All of the feed, residual feed, and fecal samples of the goats were stored at −20°C for subsequent analyses.

On day 42 before morning feeding, a 20 mL blood sample was collected from the jugular vein (at right) of each goat and stored in two 10 mL evacuated tubes without anticoagulant (KC092; 16 × 100 mm; Jiangsu Kangjie Medical Devices Co., Ltd., Taizhou, China). The evacuated tubes with blood was kept on ice for 60 min, centrifuged at 3000 × g for 20 min, and then the supernatant was collected in a 1.5 mL centrifuge tube. The serum was stored at −80°C Ultra-low temperature refrigerator for later metabolite analysis.

On day 42 before morning feeding, 80 mL of rumen fluid was collected using an oral stomach tube (Length: 1.7 m, outside diameter: 12 mm; ANSCITECH, Wuhan, China), of which the first 15–20 mL from each goat was discarded to avoid saliva contamination. The rumen fluid pH was measured immediately using a digital pH meter (S400-B; Mettler Toledo, Shanghai City, China), and the rumen fluid was strained through four layers of cheesecloth. A 10 mL rumen fluid sample was placed into conical centrifuge tubes, de-proteinizing solution (100 g metaphosphoric acid /L and 0.6 g croconic acid/L) was added at a ratio of 1:1, and VFA analysis was conducted. Next, another 10 mL rumen fluid was placed into conical centrifuge tubes, and 0.5 mmol/L hydrochloric acid solution was added at a ratio of 1:1 for ammonia-N determination. The remaining rumen fluid was stored in 25 mL centrifuge tubes at −80°C for subsequent analysis of microbial protein-N and rumen bacterial communities.

### 2.3 Chemical analyses

#### 2.3.1 Feed, residual feed, and fecal chemical composition

Feed, residual feed, and fecal samples from each goat were dried in a forced air oven at 65°C for 72 h. After drying, these samples were ground using a mill (FZ102; Beijing Ever Bright Medical Treatment Instrument Co., LTD) and passed through a 1.0 mm screen, and then nutritional composition analysis was conducted. The feed, residual feed, and fecal samples were each analyzed in triplicate, which for dry matter (DM), crude protein (CP), ash, and ether extract (EE), according to the AOAC (15) methods No. 924.45, 984.13, 942.05, and 920.00, respectively. The organic matter (OM) values were obtained by subtracting the ash concentrations from the DM concentrations of the samples. Neutral detergent fiber (NDF) and acid detergent fiber (ADF) contents were determined using an auto-fiber analyzer (F800, Hanon Advanced Technology Group Co., Ltd., Jinan City, China) following by Van Soest et al. (65) and Robertson and Van Soest (66), respectively. The NDF (exclusion of *α*-amylase and sodium sulfite) and ADF values of feed, residual feed, and fecal samples was remains residual ash.

Acid-insoluble ash (AIA) was used as an internal marker to calculate the total tract digestibility of DM, CP, OM, EE, NDF, and ADF using the following formula (16):


$$\begin{array}{l} \mathrm{Apparent total tract digestibility},\%=100–100\\ \times \left(\mathrm{nutrients in feces,}\%\right)/\left(AIA\;\mathrm{in feces,}\%\right)\\ \times \left(AIA\;\mathrm{in}\;DM\;\mathrm{consumed,}\%\right)/\left(\mathrm{nutrients consumed,}\%\right) \end{array}$$

#### 2.3.2 Rumen fermentation parameters

The VFAs concentrations of the rumen fluid, which consist of acetate, propionate, butyrate, and iso-VFAs (the sum of iso-butyrate, valerate, and iso-valerate concentrations), were measured using gas chromatography (GC) with a capillary column (AT-FFAP: length 30 m × pore size 0.32 mm × diameter 0.5 mm) using a Shimadzu 2010 plus system (Shimadzu Corporation, Kyoto, Japan) according to Liu et al. (17). Ammonia-N concentrations were measured at an absorbance of 630 nm by a SpectraMax M5 spectrometer (Molecular Devices, San Jose, CA, USA), which was following by Hristov et al. (18). The microbial protein-N (MCP) concentration was determined using a commercial Lowry’s Assay Kit (Cat No. A045-2-2, Nanjing Jiancheng Bioengineering Institute Ltd., Nanjing, China) according to the manufacturer’s instructions (19).

#### 2.3.3 Serum biochemical, antioxidant enzymes, and hormones indices

The serum total protein (TP), albumin, urea, triglyceride, *β*-Hydroxybutyric acid, glucose, catalase, total superoxide dismutase (SOD), total antioxidant capacity (T-AOC), glutathione peroxidase (GSH-PX), malondialdehyde, insulin, glucagon, growth hormone, leptin, and insulin-like growth factor-1 (IGF-1) concentrations of Leizhou goats were analyzed. Serum metabolite analysis was performed using standard commercial kits and an automatic biochemical analyzer (Kehua ZY KHB-1280; Hunan Fengrui-Biological Co., Ltd., Changsha, China) according to the manufacturer’s instructions. Globulin values were obtained by subtracting the concentration of TP from that of albumin.

#### 2.3.4 Rumen fluid DNA extraction, 16S rRNA gene amplification, and sequencing

A total of 1.00 mL rumen fluid samples were used to extract the genomic DNA of rumen bacteria using a DNA extraction kit (Product No: DP328, Tiangen Biotech, Beijing, China), according to the manufacturer’s protocol. The DNA concentration and purity were assessed using NanoDrop One (Thermo Fisher Scientific, Madison, WI, USA). The extracted DNA quality was measured using agarose gel electrophoresis (1%, Axygen Biosciences, Union City, CA, USA). Samples (purity ≥1.8) were selected for later polymerase chain reaction (PCR) processing.

The extracted DNA samples were subjected to conventional PCR amplification and bioinformatics analysis performed by Shanghai Majorbio Bio-Pharm Technology Co., Ltd. (Shanghai, China). Bacterial diversity was measured by sequencing the hypervariable regions V3–V4 of 16S rRNA, which were amplified using PCR with the primers 338F (5′-ACTCCTACGGGAGGCAGCAG-3′) and 806R (5′-GGACTACHVGGGTWTCTAAT-3′). Bacterial 16S amplification, quality filtering, clustering, and analysis of the 16S rRNA sequencing data were performed as described previously (6). The PCR amplification reaction conditions and 16S rRNA gene sequencing procedures were as follows: initial denaturation at 95°C for 180 min, followed by 30 cycles, consisting of denaturation at 95°C for 30 s, annealing at 55°C for 30 s, and extension at 72°C for 30 s, with a final extension at 72°C for 10 min and then maintenance at 10°C. The PCR experiments were performed in triplicate in 20 μL mixture, which consisted of 4 μL TransStart FastPfu buffer (5×), 2 μL deoxyribonucleotides triphosphate (dNTPs; 2.5 mM), 0.8 μL 338F and 806R primers (5 mM), 0.2 μL bovine serum albumin, 0.4 μL TransStart FastPfu DNA Polymerase of template DNA, and ddH2O was added to make the volume up to 20 μL. Agarose gel (2.0%) electrophoresis (Axygen Biosciences, Union City, CA, USA) was used to assess the success of the PCR reactions.

The purified PCR amplicon products were mixed in equimolar amounts and paired-end sequenced (2 × 300 bp) using an Illumina MiSeq PE300 platform (Illumina, San Diego, CA, USA). The sequenced was conducted by Majorbio Bio-Pharm Technology Co., Ltd. (Shanghai, China) and the sequencing data analysis was performed on the Majorbio Cloud Platform,^1^ which is available online. Based on the OTUs information, rarefaction curves and alpha diversity indices including observed OTUs, Chao1 richness, Shannon index and Good’s coverage were calculated with Mothur v1.30.1 (20). The similarity among the microbial communities in different samples was determined by principal coordinate analysis (PCoA) based on Bray–curtis dissimilarity using Vegan v2.5–3 package. The linear discriminant analysis (LDA) effect size (LEfSe)^2^ (21) was performed to identify the significantly abundant taxa (phylum to genera) of bacteria among the different groups (LDA score > 2, *p* < 0.05).

### 2.4 Statistical analysis

Data on growth performance, apparent total tract nutrient digestibilities, rumen pH, concentrations of VFAs, ammonia-N, MCP, and serum metabolite indices were statistically analyzed using *T*-TEST in SAS software (SAS 9.4; SAS Institute Inc., Cary, NC, USA). Data are presented as the means ± standard error (SEM). The statistically significant differences were considered when *p* values <0.05.

Spearmen’s rank correlation analyses were performed using the “corrplot” package in R (Version 3.6.3) to explore the relationship between the relative abundances of rumen bacteria (at genus levels, relative abundance >0.5%) and rumen pH and concentrations of VFAs, ammonia-N, and MCP parameters.

## 3 Results

### 3.1 Growth performance and apparent total tract nutrient digestibilities

**Figure 1:**
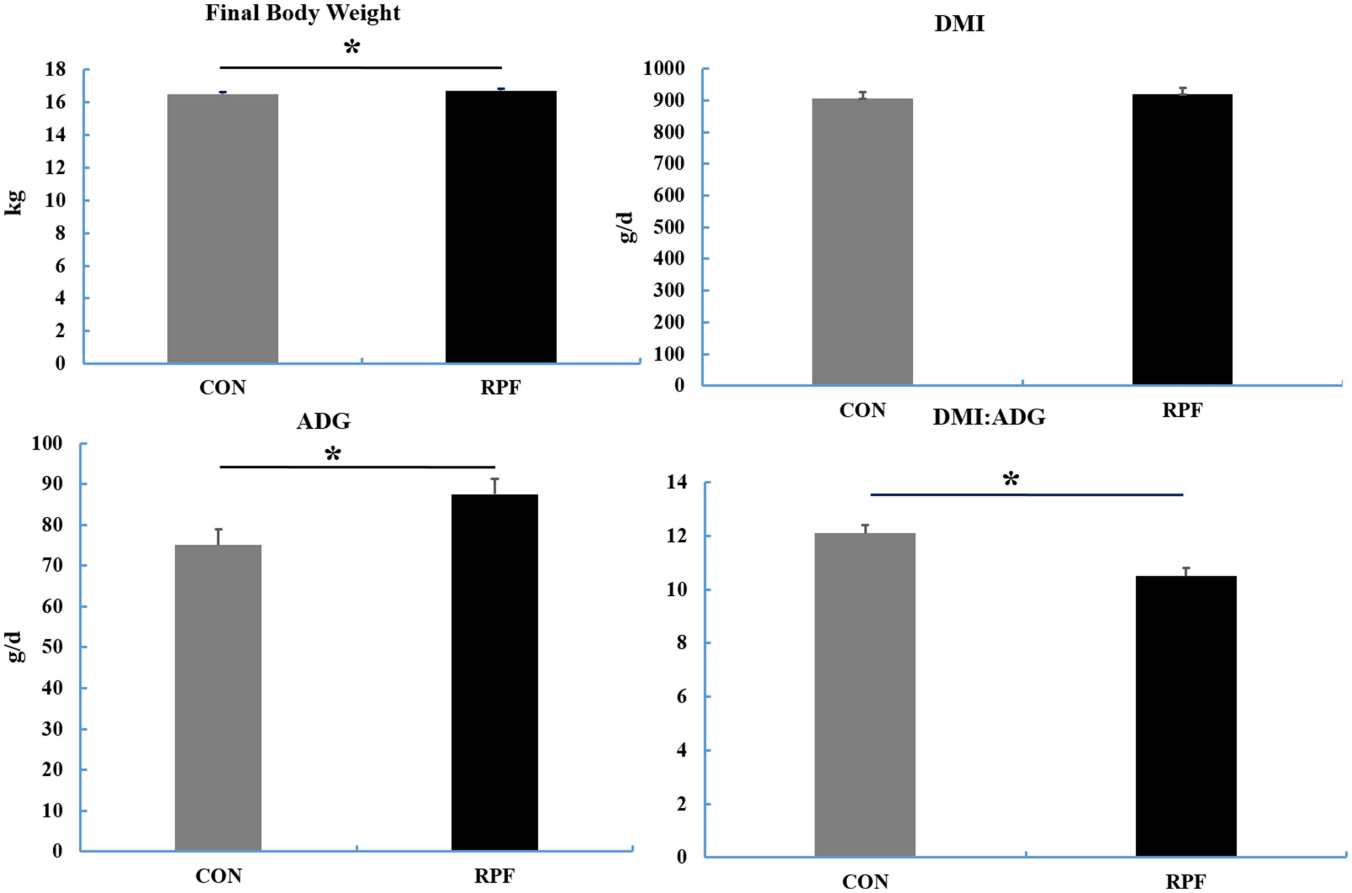
The final body weight, DMI, ADG, and the ratio of DMI to ADG in Leizhou goats between CON and RPF suppelemtnation groups. DMI, dry matter intake; ADG, average daily gain; CON, basal group without rumen protected fat supplementation; RPF, basal group with 2.4% rumen-protected fat supplementation.

The final BW and ADG were higher (*p* < 0.05) and the ratio of DMI to ADG was lower (*p* < 0.05) in the 2.4% RPF supplementation group compared with those in the CON group (Figure 1). The apparent total tract digestibility of dry matter, organic matter, crude protein, neutral and acid detergent fiber in the 2.4% RPF supplementation group was higher than that in the control group (*p* < 0.05; Table 2).

**Table 2:** Effect of rumen-protected fat on the digestibilities of dietary nutrients in Leizhou goats.

Items	CON	2.4% RPF	SEM	*p*-values
Dry matter, %	49.6	54.4	0.92	0.003
Organic matter, %	63.7	68.5	0.90	<0.01
Crude protein, %	60.1	66.6	1.13	<0.001
Ether extract, %	55.5	55.9	0.80	0.823
Neutral detergent fiber, %	44.0	46.7	0.70	0.049
Acid detergent fiber, %	29.0	34.6	1.05	0.002

CON, basal group without rumen protected fat supplementation; RPF, basal group with 2.4% rumen-protected fat supplementation; SEM, standard error of the mean.

### 3.2 Ruminal pH and concentrations of VFAs, ammonia-N, and MCP

The proportion of acetate and MCP concentrations were higher and the proportion of propionate was lower (*p* < 0.05) in the 2.4% RPF supplementation group compared with those in the control group (Table 3).

**Table 3:** Effect of rumen-protected fat on rumen fermentation parameters in Leizhou goats.

Items	CON	2.4% RPF	SEM	*p*-values
pH	6.95	7.06	0.05	0.226
Ammonia-N, mg/100 mL	13.5	13.6	0.058	0.126
MCP, mg/100 mL	6.93	7.40	0.096	<0.01
Total VFAs, mmol/L	30.9	32.9	1.22	0.993
VFA, mol/100 mol				
Acetate	65.1	67.1	0.48	0.028
Propionate	13.1	11.2	0.37	<0.01
Butyrate	13.4	13.9	0.435	0.570
Iso-VFA	8.57	7.72	0.253	0.090
Acetate: propionate	5.24	6.08	0.328	0.215

VFA, volatile fatty acid; iso-VFA, sum of iso-butyrate, valerate, and iso-valerate; MCP, microbial protein; CON, basal group without rumen protected fat supplementation; RPF, basal group with 2.4% rumen-protected fat supplementation; SEM, standard error of the mean.

### 3.3 Serum biochemical, antioxidant enzymes, and hormones indices

Serum concentrations of glucose, T-AOC, GSH-PX, glucagon, growth hormone, and IGF-1 were higher (*p* < 0.05) and those of total protein, globulin, triglyceride, malondialdehyde, insulin, and leptin levels were lower (*p* < 0.05) in the 2.4% RPF supplementation group compared with those in the control group (Table 4).

**Table 4:** Effect of rumen-protected fat on serum biochemical, antioxidant enzymes, and hormones indices in Leizhou goats.

Items	CON	2.4% RPF	SEM	*p*-values
Total protein, g/L	63.8	56.8	1.71	0.033
Albumin, g/L	38.9	36.4	0.80	0.133
Globulin, g/L	24.9	20.4	1.08	0.031
Blood urea nitrogen, mmol/L	7.96	7.54	0.17	0.236
Glucose, mmol/L	4.30	4.86	0.10	0.026
Triglyceride, mmol/L	0.61	0.36	0.06	0.014
*β*-hydroxybutyrate, mmol/L	0.14	0.14	0.01	0.843
Catalase, U/mL	2.52	2.60	0.12	0.741
Superoxide dismutase, U/mL	166	163	4.2	0.775
Total antioxidant capacity, U/mL	5.90	6.68	0.29	0.014
Malondialdehyde, nmol/mL	3.27	1.95	0.25	<0.001
Glutathione peroxidase, U/mL	119	129	4.7	0.048
Insulin, IU/mL	13.7	9.62	1.62	0.023
Glucagon, pg./mL	461	604	31.5	0.014
Leptin, ng/mL	4.73	3.19	0.32	<0.01
Growth hormone, ng/mL	0.40	0.62	0.04	<0.01
IGF-1, ng/mL	146.0	165.7	6.37	0.027

IGF-1, insulin-like growth factor-1; CON, basal group without rumen protected fat supplementation; RPF, basal group with 2.4% rumen-protected fat supplementation; SEM, standard error of the means.

### 3.4 Rumen bacterial community composition

A total of 872,798 raw sequences were obtained from the 12 rumen fluid samples; 852,210 high-quality sequences remained after quality filtration, and chimeric sequences were removed. Based on the 97% nucleotide sequence identity, a total of 1,919 OTUs were obtained from the 12 rumen fluid samples.

**Figure 2:**
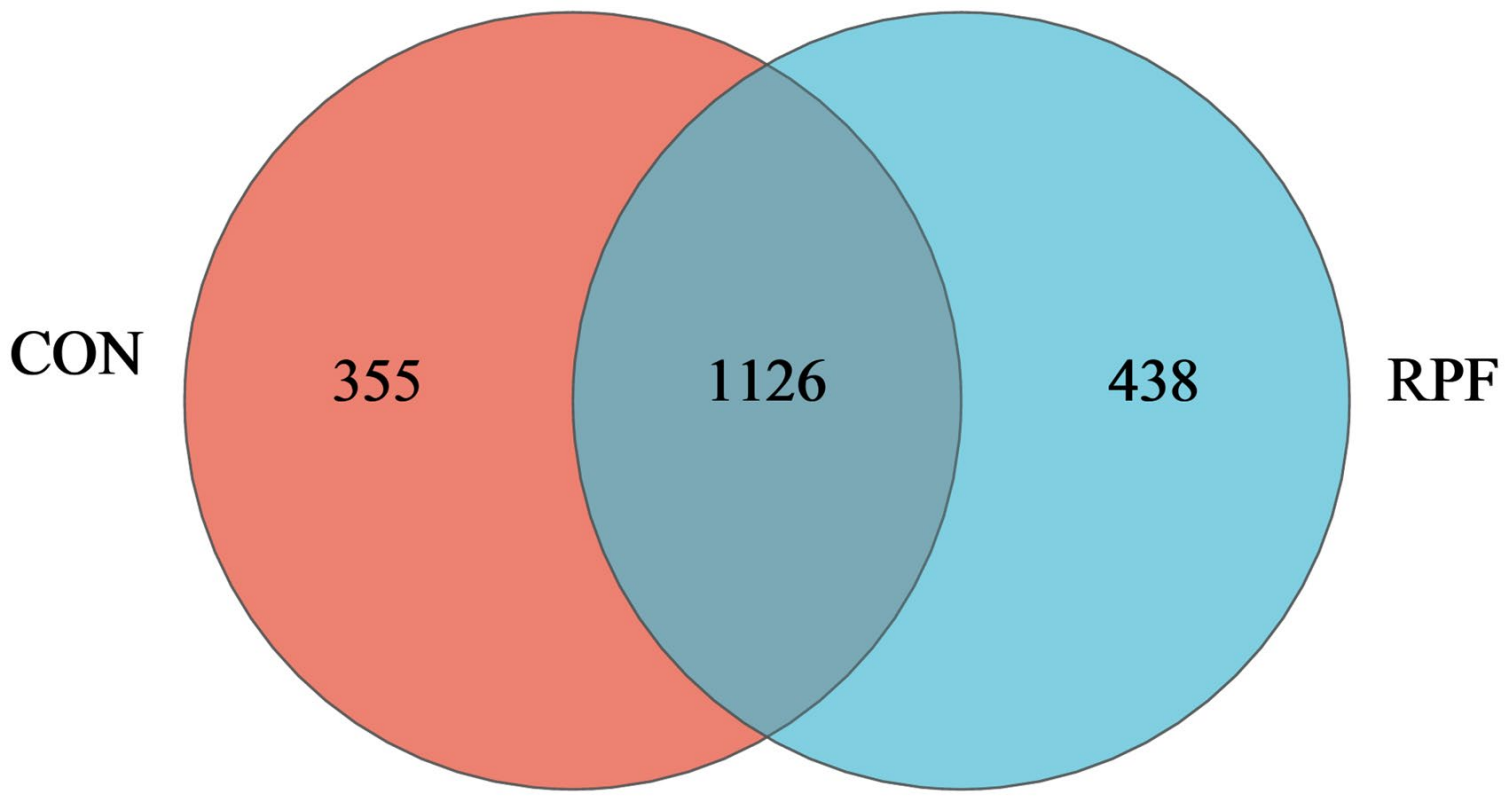
Veen plot showing different and similar OTUs in rumen fluid of Leizhou goats between CON and RPF suppelemtnation groups. CON, basal group without rumen protected fat supplementation; RPF, basal group with 2.4% rumen-protected fat supplementation.

There were 1,126 OTUs shared between these two groups, which comprised 76.0 and 72.0% of all OTUs in the control and 2.4% RPF supplementation groups, respectively (Figure 2). Specifically, there were 355 and 438 unique OTUs in the control and 2.4% RPF supplementation groups, respectively. The ACE, Chao, and Sobs indices in the 2.4% RPF supplementation group were higher than those in the control group (*p* < 0.05; Table 5). There was no significant difference (*p* > 0.05) in coverage, the Shannon index, or the Simpson index between the 2.4% RPF supplementation and control groups.

**Table 5:** Effect of rumen-protected fat on alpha diversity in the rumen fluid of Leizhou goats.

Items	CON	2.4% RPF	SEM	*p*-values
Ace	758	907	50.8	0.042
Chao	748	905	49.9	0.018
Coverage	0.998	0.998	0.0001	0.116
Shannon	4.56	4.77	0.105	0.328
Simpson	0.027	0.022	0.0027	0.370
Sobs	696	823	48.3	0.002

CON, basal group without rumen protected fat supplementation; RPF, basal group with 2.4% rumen-protected fat supplementation; SEM, standard error of the mean.

**Figure 3:**
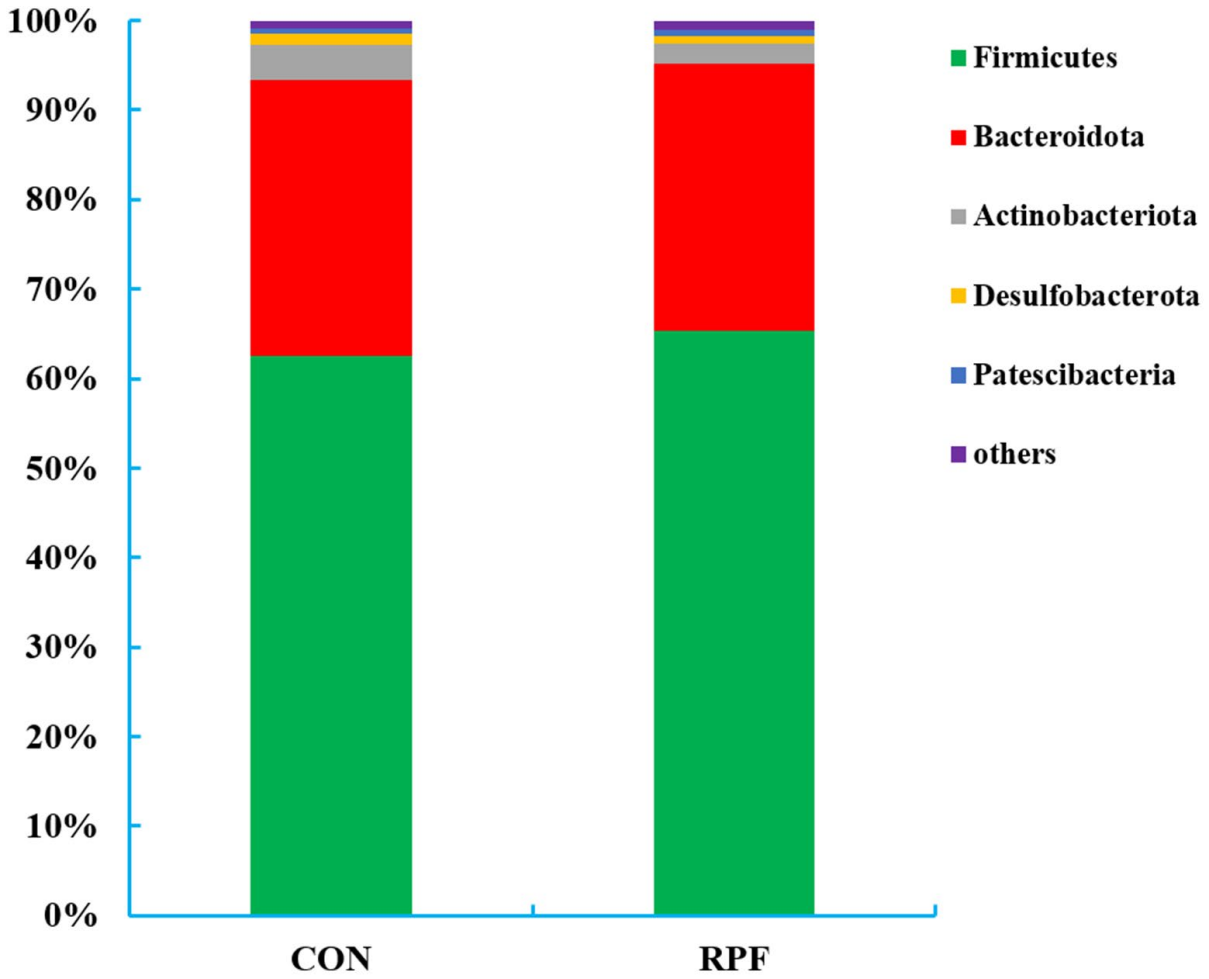
Rumen bacterial relative abundances (at the phylum level, > 1.0% of total reads) in Leizhou goats between CON and RPF supplementation groups. CON, basal group without rumen protected fat supplementation; RPF, basal group with 2.4% rumen-protected fat supplementation.

A total of 18 bacteria phyla were identified in the 12 rumen fluid samples, of which five phyla had a relative abundance above 1.0% (Figure 3; Supplementary Table S1). Firmicutes (62.5% for control group; 65.3% for 2.4% RPF supplementation group) and Bacteroidetes (30.9% for control group; 29.8% for 2.4% RPF supplementation group) were the dominant phyla, while Actinobacteria (3.83% for control group; 2.26% for 2.4% RPF supplementation group), Desulfobacterota (1.27% for control group; 0.85% for 2.4% RPF supplementation group), and Patescibacteria (0.60% for control group, 0.77% for 2.4% RPF supplementation group) were present at lower abundances. The relative abundance of Firmicutes was higher (*p* < 0.05) and that of Actinobacteria was lower (*p* < 0.05) in the 2.4% RPF supplementation group compared with those in the control group.

**Figure 4:**
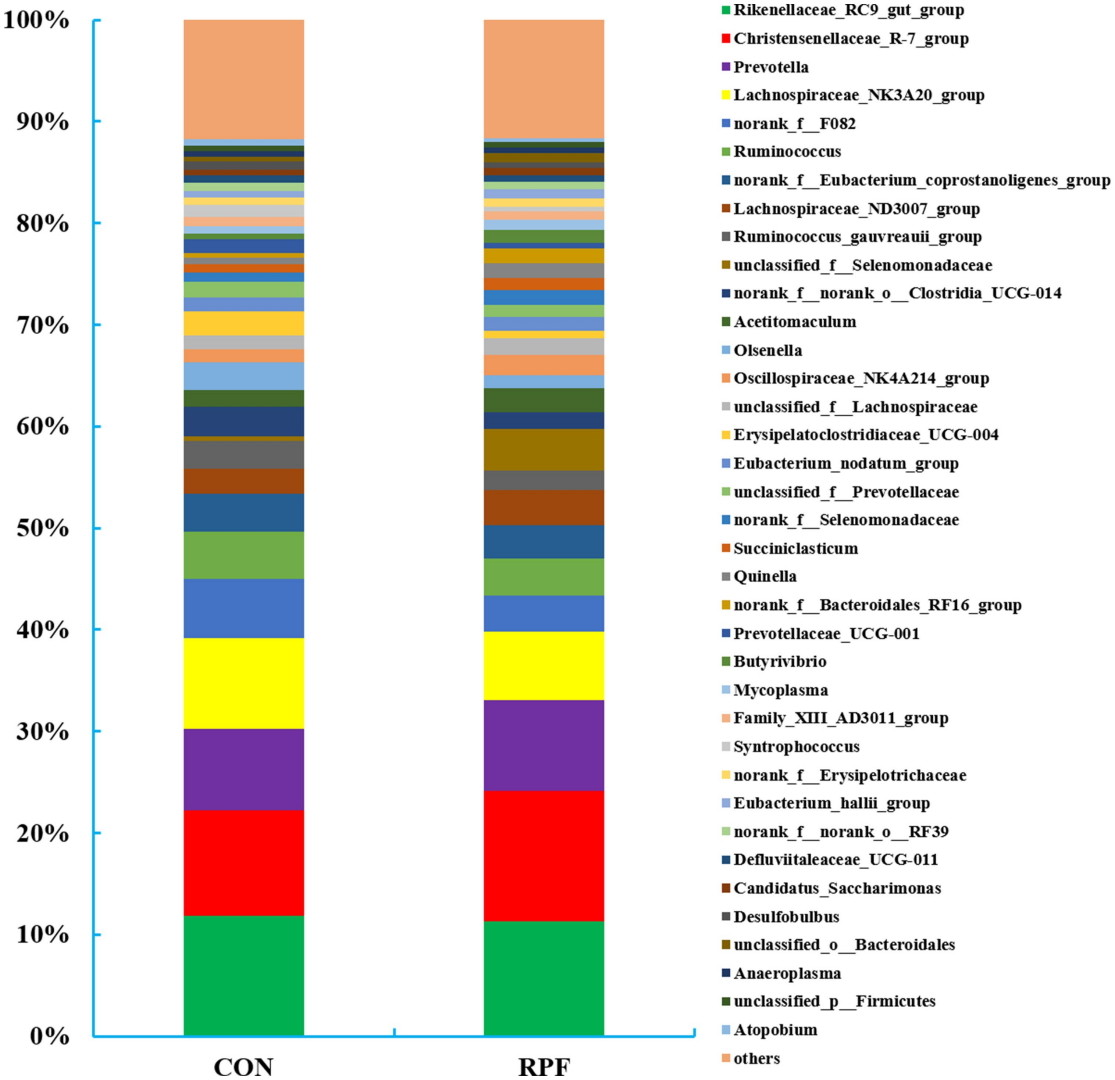
Rumen bacterial relative abundances (at the genus level, >0.5% total reads) in Leizhou goats between CON and RPF suppelemtnation groups. CON, basal group without rumen protected fat supplementation; RPF, basal group with 2.4% rumen-protected fat supplementation.

A total of 254 bacterial genera were identified in the 12 rumen fluid samples (Figure 4; Supplementary Table S2). The greatest abundant taxa were *Rikenellaceae_RC9_gut_group* (11.9% in control group; 11.3% in 2.4% RPF supplementation group), *Christensenellaceae_R-7_group* (10.4% in control group; 12.8% in 2.4% RPF supplementation group), and *Prevotella* (7.99% in control group; 8.94% in 2.4% RPF supplementation group). The relative abundances of *Christensenellaceae_R-7_group*, *unclassified_f__Selenomonadaceae*, *norank_f__Selenomonadaceae*, *Quinella*, *norank_f__Bacteroidales_RF16_group*, and *unclassified_o__Bacteroidales* were higher (*p* < 0.05), and *Lachnospiraceae_NK3A20_group*, *norank_f__F082*, *Olsenella*, *Erysipelatoclostridiaceae_UCG-004*, and *Syntrophococcus* were lower (*p* < 0.05) in the 2.4% RPF supplementation group compared with those in the control group.

**Figure 5:**
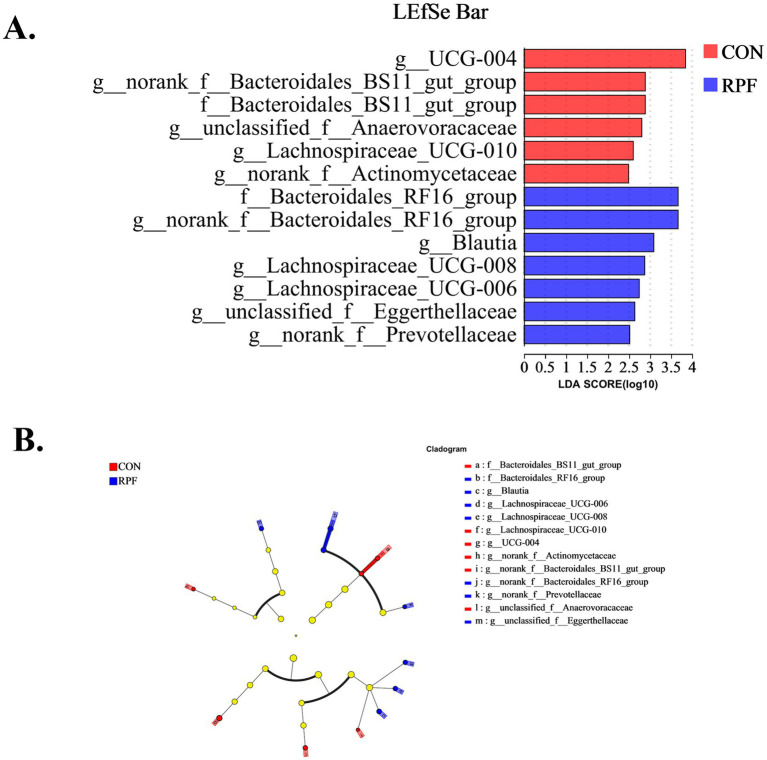
Linear discriminant analysis effect size (LEfSe) results for rumen bacteria in Leizhou goats between CON and RPF supplementation groups. **(A)** Linear discriminant analysis. **(B)** Cladogram. Prefixes represent abbreviations for the taxonomic rank of each taxon, family (f^−^), and genus (g^−^). CON, basal group without rumen protected fat supplementation; RPF, basal group with 2.4% rumen-protected fat supplementation.

Linear discriminant analysis effect size (LEfSe) was used to compare significantly different bacterial taxa at the phylum, genus, and OTU levels among the 12 rumen fluid samples from Leizhou goats (Figures 5A,B). There were 6 and 7 different taxa with a default LDA cutoff of ±2.0 in the control and 2.4% RPF supplementation groups, respectively. The bacteria biomarkers in the control group were *g_UCG-004*, *g_norank_f_Bacteroidales_BS11_gut_group*, f_Bacteroidales_BS11_gut_group, *g_unclasssified_f_Anaerovoracaceae*, *g_Lachnospiraceae_UCG-010*, and *g_norank_f_Actinomycetaceae,* and those in the 2.4% RPF supplementation group were f_Bacteroidales_RF16_group, *g_norank_f_Bacteroidales_RF16_group*, *g_Blautia*, *g_Lachnospiraceae_UCG-008*, *g_Lachnospiraceae_UCG-006*, and *g-norank_f_Prevotellaceae*.

### 3.5 Correlation between ruminal pH, the concentrations of VFAs, ammonia-N, and MCP, and bacterial community composition at genus levels

**Figure 6:**
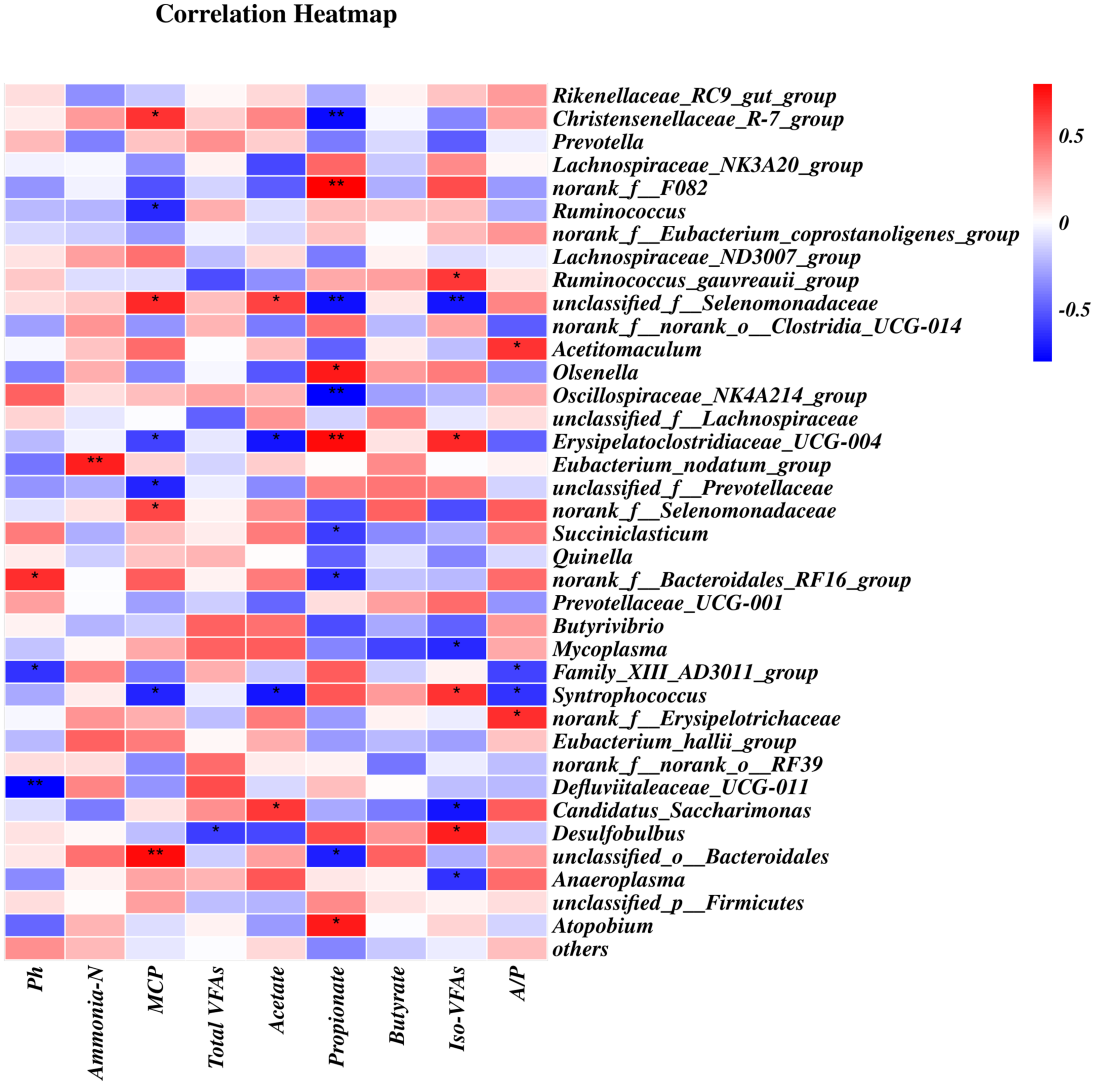
Correlations between rumen bacterial relative abundance at the genus-level (>0.5% total read) and rumen pH, VFAs, MCP, and ammonia-N concentrations.

Spearman’s rank correlation analysis was showed numerous significant correlations between the relative abundances of the bacterial composition at genus levels (> 0.5%) and ruminal pH and the concentrations of VFAs, ammonia-N, and MCP (Figure 6). A total of 18 positive (*p* < 0.05) and 21 negative (*p* < 0.05) correlations was identified. The *Christensenellaceae_R-7_group* and *unclassfied_o_Bacteroidales* were positively correlated with MCP concentration but negatively correlated with propionate concentration. *Norank_f_F082*, *Olsenella*, and *Atopobium* were positively correlated with propionate concentration whereas *Oscillospiraceae_NK4A214_group* and *Succiniclasticum* were negatively correlated with propionate concentration. *Ruminococcus* was negatively correlated with MCP concentration. *Unclassified_f_Selenomadaceae* was positively correlated with the MCP and acetate concentrations but negatively correlated with propionate and iso-VFA concentrations. *Erysipelatoclostridiaceae_UCG-004* was positively correlated with propionate and iso-VFA concentrations, but negatively correlated with MCP and acetate concentrations. The *Ruminococcus-gauvreauii_group* was positively correlated with iso-VFA concentration, whereas *Mycoplasma* and *Anaeroplasma* were negatively correlated with iso-VFA concentration.

## 4 Discussion

### 4.1 Effect of 2.4% rumen protected fat supplementation on DMI, ADG, and nutrient apparent digestibilities in Leizhou goats

Previous studies investigating the effects of RPF have reported contrasting results. Some studies have reported that RPF supplementation does not influence ADG in Awassi lambs (22), dairy cows (23), or Dorper sheep (4). In contrast, RPF enhances ADG in culled ewes (24) and finishing goats (11). In the current study, the ADG of goats in the 2.4% RPF supplementation group was higher than that in the control group, as reported in a previous study on finishing goats consumed RPF (10). The greater ADG in goats fed the RPF diets was probably due to the extra energy supply from RPF. The rumen bypass of the rumen protected fat in the current study was ≥825 g/kg (data from supplier), which could indicate that a part of the rumen protected fat was released into the rumen. In addition, methane energy production in the 2.4% RPF supplementation group was lower than that in the control group, likely because fat can reduce methane production in dairy Alpine goats (25), dairy cows (26), and growing Hanwoo steers (67). Interestingly, we found that the abundances of H_2_ incorporating bacteria in rumen fluid, for example, *Quinella* (27), were higher in the 2.4% RPF supplementation group compared with those in the control group.

RPF does not influence DM, OM, CP, NDF, or ADF digestibility in Dorper sheep (4), crossbred cows (28), or buffaloes (29). In contrast, previous studies reported increased OM and CP digestibility in cull ewes (30) and lactating dairy cows (31), respectively, following RPF supplementation. In the current study, the total apparent digestibility of DM, OM, CP, NDF, and ADF in the 2.4% RPF supplementation group was higher than that in the control group. A previous study reported an increase in *Fibrobacter succinogenes, Ruminococcus albus*, and total cellulolytic bacteria abundances in the fat-fed group compared to those in the control group, which could explain the higher NDF and ADF digestibilities in the 2.4% RPF supplementation group compared to those in the control group (4). Moreover, the relative abundance of fibrolytic bacteria, such as *Christensenellaceae_R-7_group* (27) and *norank_f__Bacteroidales _RF16_group* (32), in the rumen fluid was higher in the 2.4% RPF supplementation group compared with those in the control group. Behan et al. (4) reported an increase in EE digestibility in the RPF group, which could be ascribed to the inclusion of high-quality fat in the diet. However, in the present study, there was no significant difference in the digestibility of EEs between the 2.4% RPF supplementation and control groups. The differences in the fatty acid composition of the RPF could explain this.

### 4.2 Effect of 2.4% rumen protected fat supplementation on ruminal pH and the concentration of VFAs, ammonia-N, and MCP in Leizhou goats

In the present study, the ruminal pH ranged from 6.95 to 7.06 for all goats, which is within the optimal range of 6.2 to 7.2 for ruminants (33). The pH is not affected by rumen-protected products (34), lysine, or methionine (5, 35). However, ruminal pH increases following 2.4% RPF supplementation in goats (4) and beef steers (36). This variation in pH may be due to the different fat forms applied in these animal species. The concentration of ammonia-N, as the major N source for MCP synthesis, was 13.5 and 13.6 mg/100 mL in the CON and RPF groups, respectively, which were within the optimal range of 5 to 25 mg/100 mL for ruminants (27). Moreover, the MCP concentration was higher in the 2.4% RPF supplementation group compared with that in the control group, which was probably due to increased energy levels enhancing microbial activity when the goats consumed rumen-protected products (5).

Ruminal VFAs provide more than 70% of the energy available to ruminants, which is generated mainly from dietary carbohydrates. In the current study, the VFA levels showed no differ between the control and 2.4% RPF supplementation groups. The same results were reported in a previous study, where neither source nor levels of RPF affected total VFA and the ratio of acetate to propionate in steers that consumed calcium soaps and hydrogenated animal fats (37). Acetate production is related to fiber digestion and fibrolytic bacteria abundance (38). In support of this, in the current study, the proportion of acetate increased while that of propionate decreased as fiber digestibility increased in the RPF group compared with those in the control group. Additionally, ruminal fibrolytic bacteria were more abundant in the 2.4% RPF supplementation group than in the control group.

### 4.3 Effect of 2.4% rumen protected fat supplementation on serum biochemical, antioxidant enzymes, and hormones indices in Leizhou goats

Serum metabolite indices could reflect the metabolic and health statuses of goats. A previous study reported that total serum protein concentration is not affected by dietary canola and palm oil blend supplementation (39). Additionally, another study reported that total serum protein concentration ranges from 60 to 75 g/L in goats (40). In the current study, the total serum protein concentration was 63.8 and 56.8 g/L in the control and 2.4% RPF supplementation groups, respectively. This value was within or slightly below the normal range. In addition, total protein concentration was lower in the RPF group compared with that in the control group. Serum total protein is mainly affected by animal species, age, and dietary composition (41). Studying the ability of dietary fat levels to stimulate glucagon secretion are very complex because fat is found in numerous forms, and their stimulatory effects may vary (42). The serum concentration of triglyceride was decreased, which could explain by emulsified by bile salts in the intestine and then intestinal lipases degrade TGL into FA, mono, di-acyltriglycerol, and glycerol, decreasing their circulating pool (43, 44).

In the current study, the serum glucagon and glucose concentrations were higher and serum insulin concentrations were lower in the 2.4% RPF supplementation group compared with those in the control group. This could be explained by glucagon being released to help release glucose into the blood when a high-fat diet is consumed (29). The T-AOC capacity reflects the ability of antioxidants to remove harmful free radicals from blood and cells. Malondialdehyde is regarded as the final product of polyunsaturated fatty acid peroxidation. A previous study found no difference in serum antioxidant levels in goats consuming canola and palm oil blends (39). In the present study, T-AOC and GSH-PX activity were higher in the 2.4% RPF supplementation group than that in the control group, and the concentration of malondialdehyde was lower in the 2.4% RPF supplementation group than that in the control group.

Growth hormone and IGF-1 promote growth (45). The serum concentrations of growth hormone and IGF-1 were higher in the 2.4% RPF supplementation group compared with those in the control group, which was in agreement with the ADG changes between the two groups. A previous study reported that the serum concentrations of glucose and glucagon were improved, whereas the insulin was decreased in yaks when they consumed dietary varying different energy levels (33). In the current study, the concentrations of glucose and glucagon were improved, whereas the insulin was decreased, which could explain that the RPF could provide energy to Leizhou Goats. Our results showed that the serum concentration of leptin was decreased in Leizhou goats when consumed the RPF, which in agreement with previous studies in beef cattle (9).

### 4.4 Effect of 2.4% rumen protected fat supplementation on rumen bacterial community composition in Leizhou goats

The diversity and stability of rumen microbes play vital roles for the host. Typically, a greater rumen microbial diversity was promotes the stability of the bacterial community in the rumen (35). Diversity indices are influenced by the animal species’ age (46) and dietary composition (47). In the present study, the ACE, Chao 1, and Sobs indices were in the 2.4% RPF supplementation group were higher than those in the CON, which indicated a higher rumen bacterial richness and diversity in goats that consumed RPF. This suggested that although the number of potential species in the sample (indicating increased richness) increased, the abundance of these new species remains relatively low, thus having minimal impact on overall diversity. The Shannon and Simpson indices account not only for species count but also for the evenness in species abundance distribution. When the proportion of newly introduced species is very low, evenness remains largely unchanged, leading to minimal or no significant change in the Shannon and Simpson indices.

As reported in lactating Tianzhu White yaks (17), Heifers (48), Chinese Mongolian sheep and Dorper × Chinese Mongolian crossbred sheep (49), and Murciano-Granadina goats (50), we found that Bacteroidetes and Firmicutes were the dominant phyla in the rumen fluid for Leizhou goat. Firmicutes are involved to degrade the cellulose, hemicellulose, starch, and oligosaccharides. Our results observed that the fiber digestibility was greater in the 2.4% RPF supplementation group compared with that in the control group, a higher relative abundance of Firmicutes in the rumen fluid in the RPF group than in the control group was expected. Actinobacteria, a phylum of gram-positive bacteria, is commonly found in ruminants. This bacterial phylum plays an important role in the fermentation of plant materials and production of volatile fatty acids, which are the main energy sources for the host animal. In the current study, the relative abundance of Actinobacteria was lower in the 2.4% RPF supplementation group compared with that in the control group, which is in agreement with the findings of a previous report showing that the abundance of Actinobacteria decreased with increasing energy levels in Holstein heifers (51).

At the genus level, the greatest dominant rumen bacteria were *Rikenellaceae_RC9_gut_group*, and then followed by *Christensenellaceae_R-7_group,* and *Prevotella*. The top three dominant rumen bacteria genera were *Prevotella 1*, *Ruminococcaceae NK4A214 group*, and *Christensenellaceae R-7 group* (52) in Lezhi black goats, whereas *Prevotella, norank_f_F082,* and *Ruminococcus* were dominant in Guanzhong goats (53). This difference could be explained by the animal breed and dietary composition. In addition, we found that the abundance of *Christensenellaceae_R-7_group* was higher in the 2.4% RPF supplementation group than that in the control group, which could explain why this genus enhanced and boosted food absorption and digestion, as well as the host ADG (54, 55).

A previous study reported that the relative abundance of *Selenomonadaceae* was higher in the rumens of dairy cows with high nitrogen utilization efficiency (56). In the current study, the *unclassified_f__Selenomonadaceae* and *norank_f__Selenomonadaceae* were more abundant in the 2.4% RPF supplementation group than in the control group, which could explain the higher crude protein digestibility observed in the RPF group compared with that in the control group. The abundance of *Lachnospiraceae_NK3A20_group*, an H_2_-producing bacterium, was lower in the RPF group than that in the control group, which could be explained by the decrease in methane production in Holstein-Friesian dairy cows fed RPF (57). *Norank_f__F082*, an unclassified genus of Bacteroidetes is widely distributed in the rumen and is mainly involved in carbohydrate degradation (7, 58). Moreover, the relative abundance of *norank_f__F082* was positively correlated with propionate concentration (59). In the present study, the relative abundance of *norank_f_F082* was lower in the 2.4% RPF supplementation group than in the control group and was positively correlated with the concentration of propionate. *Olsenella*, a gram-negative anaerobic bacterium, belongs to the family of *Lachnospiraceae* and is a lactate and succinate producer (60). Succinate is a precursor to propionate (61). In the present study, the propionate levels were lower in the 2.4% RPF supplementation group than in the control group, which could be partially explained by the higher relative abundance of *Olsenella* in the control group.

### 4.5 Effect of 2.4% rumen protected fat supplementation on the correlation between ruminal pH, the concentrations of VFAs, ammonia-N, and MCP; and bacterial community composition in Leizhou goats

*Christensenellaceae_R-7_group*, which belongs to the Christensenellaceae family, was positively correlated with protein metabolism and the levels of the intestinal metabolites of dietary proteins in animal production (26, 62). Our results showed that the abundance of *Christensenellaceae_R-7_group* was positively correlated with MCP concentration, which is in agreement with the results of a study by An et al. (63). *Candidatus_Saccharimonas* abundance was positively correlated with acetate levels, which could be because the members of the genus *Candidatus_Saccharimonas* mainly produce acetate (64). In future research, clarification of the relationship between ruminal bacterial community composition and function and host production is needed.

## 5 Conclusion

We found that RPF supplementation resulted in increased ADG and a decreased ratio of DM intake compared to those of the control group in Leizhou goats. The digestibilities of DM, OM, CP, NDF, and ADF were higher in the 2.4% RPF supplementation group than in the control group. Moreover, the ruminal bacterial communities were altered in goats fed RPF. In conclusion, supplementation with 2.4% RPF can improve the ADG by regulating the rumen bacterial communities and enhancing the nutrient digestibility and serum antioxidant indices in Leizhou goats.

## Data Availability

The data presented in this study are deposited in the NCBI REPOSITORY, accession number PRJNA1184368. The sequencing raw data were deposited in the NCBI BioProject database under the accession number PRJNA1184368.

## Footnotes

^1^ www.Majorbio.com

^2^ http://huttenhower.sph.harvard.edu/LEfSe
