# Supplementary material for: Comparison of average daily gain, apparent digestibility, rumen fermentation parameters and bacterial communities, and serum antioxidant indices in Leizhou goats fed with or without rumen-protected fat
Source: Front Vet Sci. 2024 Dec 23;11:1518826. doi: 10.3389/fvets.2024.1518826 (PMC11700983; doi:10.3389/fvets.2024.1518826)
Supplement: Supplementary file 1 [file Table_1.DOCX]

STable 1 The ruminal bacterial communities at phylum levels (>0.5%) in response to dietary rumen-protected fat in goat rumen fluid.

| Items | CON | RPF | SEM | P-values |
| --- | --- | --- | --- | --- |
| Firmicutes | 62.5 | 65.3 | 0.77 | 0.046 |
| Bacteroidota | 30.9 | 29.8 | 0.57 | 0.355 |
| Actinobacteriota | 3.83 | 2.26 | 0.363 | 0.022 |
| Desulfobacterota | 1.27 | 0.85 | 0.166 | 0.219 |
| Patescibacteria | 0.60 | 0.77 | 0.114 | 0.479 |
| others | 0.88 | 0.98 | 0.102 | 0.624 |
| F:B | 2.03 | 2.21 | 0.064 | 0.157 |

STable 2 The ruminal bacterial communities at genus levels (>0.5%) in response to dietary rumen-protected fat in goat rumen fluid.

| Items | CON | RPF | SEM | P-values |
| --- | --- | --- | --- | --- |
| *Rikenellaceae_RC9_gut_group* | 11.9 | 11.3 | 0.56 | 0.653 |
| *Christensenellaceae_R-7_group* | 10.4 | 12.8 | 0.51 | 0.010 |
| *Prevotella* | 7.99 | 8.94 | 0.322 | 0.146 |
| *Lachnospiraceae_NK3A20_group* | 8.95 | 6.75 | 0.468 | 0.010 |
| *norank_f__F082* | 5.77 | 3.54 | 0.398 | 0.001 |
| *Ruminococcus* | 4.62 | 3.63 | 0.291 | 0.088 |
| *norank_f__Eubacterium_coprostanoligenes_group* | 3.77 | 3.31 | 0.127 | 0.069 |
| *Lachnospiraceae_ND3007_group* | 2.44 | 3.38 | 0.271 | 0.079 |
| *Ruminococcus_gauvreauii_group* | 2.75 | 1.96 | 0.295 | 0.191 |
| *unclassified_f__Selenomonadaceae* | 0.46 | 4.10 | 0.701 | 0.003 |
| *norank_f__norank_o__Clostridia_UCG-014* | 2.90 | 1.63 | 0.460 | 0.179 |
| *Acetitomaculum* | 1.69 | 2.40 | 0.246 | 0.156 |
| *Olsenella* | 2.74 | 1.28 | 0.341 | 0.023 |
| *Oscillospiraceae_NK4A214_group* | 1.20 | 1.94 | 0.206 | 0.067 |
| *unclassified_f__Lachnospiraceae* | 1.44 | 1.67 | 0.130 | 0.419 |
| *Erysipelatoclostridiaceae_UCG-004* | 2.31 | 0.76 | 0.284 | 0.001 |
| *Eubacterium_nodatum_group* | 1.43 | 1.35 | 0.175 | 0.832 |
| *unclassified_f__Prevotellaceae* | 1.48 | 1.18 | 0.081 | 0.057 |
| *norank_f__Selenomonadaceae* | 0.96 | 1.45 | 0.112 | 0.021 |
| *Succiniclasticum* | 0.82 | 1.21 | 0.161 | 0.243 |
| *Quinella* | 0.58 | 1.41 | 0.222 | 0.046 |
| *norank_f__Bacteroidales_RF16_group* | 0.49 | 1.44 | 0.250 | 0.041 |
| *Prevotellaceae_UCG-001* | 1.34 | 0.58 | 0.283 | 0.195 |
| *Butyrivibrio* | 0.52 | 1.29 | 0.286 | 0.190 |
| *Mycoplasma* | 0.73 | 1.02 | 0.090 | 0.110 |
| *Family_XIII_AD3011_group* | 0.96 | 0.76 | 0.089 | 0.284 |
| *Syntrophococcus* | 1.18 | 0.51 | 0.143 | 0.010 |
| *norank_f__Erysipelotrichaceae* | 0.73 | 0.82 | 0.055 | 0.439 |
| *Eubacterium_hallii_group* | 0.64 | 0.90 | 0.088 | 0.129 |
| *norank_f__norank_o__RF39* | 0.77 | 0.70 | 0.060 | 0.575 |
| *Defluviitaleaceae_UCG-011* | 0.75 | 0.63 | 0.069 | 0.427 |
| *Candidatus_Saccharimonas* | 0.60 | 0.77 | 0.075 | 0.269 |
| *Desulfobulbus* | 0.84 | 0.49 | 0.124 | 0.164 |
| *unclassified_o__Bacteroidales* | 0.38 | 0.90 | 0.102 | 0.004 |
| *Anaeroplasma* | 0.55 | 0.62 | 0.092 | 0.742 |
| *unclassified_p__Firmicutes* | 0.59 | 0.52 | 0.073 | 0.669 |
| *Atopobium* | 0.66 | 0.38 | 0.074 | 0.052 |
| *others* | 11.7 | 11.6 | 0.61 | 0.959 |
